# Loci for prediction of penicillin and tetracycline susceptibility in *Neisseria gonorrhoeae*: a genome-wide association study

**DOI:** 10.1016/S2666-5247(22)00034-9

**Published:** 2022-03-24

**Authors:** Tatum D Mortimer, Jessica J Zhang, Kevin C Ma, Yonatan H Grad

**Affiliations:** Department of Immunology and Infectious Diseases, Harvard T H Chan School of Public Health, Boston, MA, USA (T D Mortimer PhD, J J Zhang AB, K C Ma AB, Y H Grad MD); Division of Infectious Diseases, Brigham and Women’s Hospital, Harvard Medical School, Boston, MA, USA (Y H Grad)

## Abstract

**Background:**

*Neisseria gonorrhoeae* poses an urgent public health threat because of increasing antimicrobial resistance; however, much of the circulating population remains susceptible to historical treatment regimens. Point-of-care diagnostics that report susceptibility could allow for reintroduction of these regimens, but development of such diagnostics has been restricted to ciprofloxacin, for which susceptibility can be predicted from a single locus. We aimed to define genetic variants associated with susceptibility to penicillin and tetracycline.

**Methods:**

We collected publicly available global whole-genome sequencing data (n=12 045) from clinical *N gonorrhoeae* isolates, with phenotypic resistance data for penicillin (n=6935), and tetracycline (n=5727). Using conditional genome-wide association studies, we defined genetic variants associated with susceptibility to penicillin and tetracycline. We excluded isolates that could not be classified as either susceptible or resistant. To validate our results, we assembled 1479 genomes from the US Centers for Disease Control and Prevention (CDC)’s Gonococcal Isolate Surveillance Project, for which urethral specimens are collected at sentinel surveillance sites across the USA. We evaluated the sensitivity and specificity of susceptibility-associated alleles using Clinical & Laboratory Standards Institute breakpoints for susceptibility and non-resistance in both the global and validation datasets.

**Findings:**

In our conditional penicillin genome-wide association study, the presence of a genetic variant defined by a non-mosaic *penA* allele without an insertion at codon 345 was associated with penicillin susceptibility and had the highest negative effect size (*β*) of significant variants (p=5·0×10^–14^, β –2·5). In combination with the absence of *bla*_TEM_, this variant predicted penicillin susceptibility with high specificity (99·8%) and modest sensitivity (36·7%). For tetracycline, the wildtype allele at *rpsJ* codon 57, encoding valine, was associated with tetracycline susceptibility (p=5·6×10^–16^, β –1·6) after conditioning on the presence of *tetM*. The combination of *rpsJ* codon 57 allele and *tetM* absence predicted tetracycline susceptibility with high specificity (97·2%) and sensitivity (88·7%).

**Interpretation:**

As few as two genetic loci can predict susceptibility to penicillin and tetracycline in *N gonorrhoeae* with high specificity. Molecular point-of-care diagnostics targeting these loci have the potential to increase available treatments for gonorrhoea.

**Funding:**

National Institute of Allergy and Infectious Diseases, the National Science Foundation, and the Smith Family Foundation.

## Introduction

Gonorrhoea, caused by infection with *Neisseria gonorrhoeae*, is the second most reported notifiable infection in the USA—accounting for 188·4 cases per 100 000 people in 2019—and increasing antibiotic resistance has made it an urgent public health threat.^1^ Treatment is empiric, and resistance has restricted the recommended treatment in the USA to ceftriaxone, an extended spectrum cephalosporin.^2^

Despite the emergence of multidrug resistant strains,^3^ a large fraction of clinical isolates remain susceptible to multiple antibiotics.^1^ Data from the Gonococcal Isolate Surveillance Project (GISP), which is the US Centers for Disease Control and Prevention (CDC)’s sentinel surveillance system for antibiotic resistance in *N gonorrhoeae*, reported that, in 2019, 44·5% of clinical isolates were not resistant to any tested antibiotics—defined as minimum inhibitory concentrations (MICs) in the susceptible or intermediate categories. Specifically, 64·6% were non-resistant to ciprofloxacin (MIC <1 μg/mL), 72·2% were non-resistant to tetracycline (MIC <2 μg/mL), and 87·2% were non-resistant to penicillin (MIC <2 μg/mL).^1^

Point-of-care diagnostics that inform on antibiotic susceptibility might help to forestall the emergence and spread of resistance by enabling a shift from empiric to tailored treatment and expanding the number of antibiotics used to treat *N gonorrhoeae* infections.^4^ The observation that ciprofloxacin susceptibility can be predicted with high specificity and sensitivity based on *gyrA* codon 91 has led to the development of molecular tests that query this locus; the SpeeDx ResistancePlus GC, for example, was recently approved for clinical use in Europe and granted breakthrough designation by the US Food and Drug Administration.^5^ However, expansion of this sequence-based approach to other antibiotics has been hindered by the absence of single locus determinants of susceptibility and resistance.

Penicillin and tetracycline were the recommended therapies for gonorrhoea until the 1980s, when the prevalence of high-level resistance increased enough to prompt a switch in the empiric treatment regimen.^6,7^ Resistance to penicillin and tetracycline can be both chromosomal and plasmid mediated. Chromosomally-encoded resistance arises from mutations modifying the antibiotic targets—*rpsJ*^8^ for tetracycline resistance and *penA*^9,10^ and *ponA*^11^ for penicillin—and mutations in the porin *porB* and in the efflux pump *mtr* operon.^12^ The plasmid-borne β-lactamase *bla*_TEM_ confers high-level penicillin resistance and the ribosome protection protein *tetM* confers tetracycline resistance.^13,14^ Despite previously being first-line gonorrhoea treatments for decades, molecular diagnostics for penicillin and tetracycline susceptibility have been less commonly studied. Proposed diagnostics or targets of molecular surveillance for penicillin susceptibility have focused on (1) *bla*_TEM_,^15^ which performs poorly in the setting of chromosomally-encoded resistance; (2) *porB,*^16^ which neglects important target modifying mutations in *penA*; or (3) resistance-associated *penA* alleles,^17^ rather than susceptibility-associated alleles. Similarly, assays targeting *tetM* have been developed, but they have not incorporated chromosomally-encoded tetracycline resistance.^15^

Although there are multiple pathways to resistance for each drug, the key goal for sequence-based diagnostics is to predict susceptibility—rather than resistance—with high specificity. Therefore, we aimed to identify a concise set of loci that are associated with penicillin and tetracycline susceptibility using genome-wide association studies (GWAS), and to evaluate their predictive performance in gonococcal clinical isolates.

## Methods

### Study design and datasets

We collected publicly available whole-genome sequencing data (n=12 045), penicillin MICs (n=6935), and tetracycline MICs (n=5727) from clinical *N gonorrhoeae* isolates. For 2116 isolates, tetracycline MICs were reported as less than 4 μg/mL or less than 8 μg/mL. These MICs were excluded from further analyses, since we could not classify them as susceptible or resistant. To validate our results, we assembled 1479 genomes from CDC’s 2018 GISP collection,^18^ representing the first five viable isolates collected each month in 2018 from urethral specimens at sentinel surveillance sites in 32 jurisdictions across the USA. Patient characteristics, including sexual behaviour and race or ethnicity, were also reported.

We used publicly available data and did not require institutional review board approval.

### Procedures

Pipelines for genome assembly and resistance-associated allele calling are given in the appendix (pp 2, 5, 8) and follow previously described methods.^19^

### Statistical analysis

To identify variants associated with penicillin and tetracycline susceptibility, we performed conditional GWAS^20^ incorporating the presence of high effect size plasmid-mediated resistance (appendix pp 2–4). The GWAS employed a linear mixed model and were run using pyseer (version 1.2.0)^21^ with default allele frequency filters using unitigs—which are unique sequences representing single-nucleotide polymorphisms, insertions, deletions, and changes in gene content—as genetic variants.^22^ We also repeated the GWAS with k-mers as genetic variants to ensure that the unitig calling procedure did not affect our results. Most datasets reported penicillin MICs within the range of 0·06–32 μg/mL. Isolates with penicillin MICs reported imprecisely as greater than 4 μg/mL or greater than 2 μg/mL were not included in the GWAS analysis because the precise MIC was unknown; the final penicillin GWAS dataset size was 6220 isolates after excluding isolates with missing genotypic or phenotypic data. Similarly, isolates with imprecise tetracycline MICs were excluded (eg, ≤4 μg/mL or ≤8 μg/mL); the final dataset size for the tetracycline GWAS was 3453 isolates after excluding isolates with missing genotypic or phenotypic data. The GWAS incorporated isolate dataset of origin, country of origin, and presence of plasmid-encoded resistance determinants (*bla*_TEM_, *tetM*) as fixed effect covariates. A similarity matrix was included as a random effect to correct for population structure.

The significance of variants was assessed using a likelihood ratio test. We also corrected for multiple hypothesis testing using a Bonferroni correction based on the number of unique presence or absence patterns for unitigs or k-mers. The threshold for significance in the penicillin GWAS was 3·13 × 10^−7^ for unitigs and 3·49 × 10^−8^ for k-mers, and the threshold for significance in the tetracycline GWAS was 3·41 × 10^−7^ for unitigs and 4·44 × 10^−8^ for k-mers.

To predict penicillin and tetracycline susceptibility, we evaluated the sensitivity and specificity of susceptibility-associated alleles using Clinical & Laboratory Standards Institute (CLSI) breakpoints for susceptibility (penicillin MIC ≤0·06 μg/mL, tetracycline MIC ≤0·25 μg/mL) and non-resistance (susceptible or intermediate, penicillin MIC <2 μg/mL, tetracycline MIC <2 μg/mL) in both the global and validation datasets. We also used isolate metadata from the 2018 GISP collection to estimate the prevalence of isolates with susceptibility-associated genotypes across patient groups (eg, sexual behaviour and race or ethnicity). χ^2^ tests were performed in R (version 4.0.3)^23^ using infer (version 0.5.4) using a threshold for significance of p<0·05. Confidence intervals for sensitivity and specificity were calculated using the formula:

p^±1·96√p^(1−p^)n

Where $\hat{p}$ is sensitivity or specificity and *n* is the number of true positives or true negatives, respectively.^24^

### Role of the funding source

The funder of the study had no role in study design, data collection, data analysis, data interpretation, or writing of the report.

## Results

By use of conditional GWAS to identify additional variants contributing to penicillin and tetracycline susceptibility—focused on significant variants associated with increased susceptibility (ie, negative effect size β)—we found that a unitig (*penA*_01; appendix p 6) corresponding to non-mosaic *penA* alleles without the resistance-associated insertion at codon 345 was significantly associated with penicillin susceptibility (appendix p 7, p=5·0 × 10^−14^, β −2·5). After conditioning on the presence of *tetM*, we found that a unitig (appendix p 6) corresponding to the wildtype allele at *rpsJ* codon 57, encoding valine, was significantly associated with tetracycline susceptibility (appendix p 7, p=5·6 × 10^−16^, β −1·6). Significant unitigs also mapped to *porB* (penicillin p=2·0 × 10^−23^, β −0·60; tetracycline p=2·5 × 10^−50^ β −0·49) and a loss of function variant in *mtrC* (penicillin p=2·5 × 10^−50^, β −1·2; tetracycline p=1·1 × 10^−14^, β −1·0) for both antibiotics; however, effect sizes (β) were lower than unitigs mapping to antibiotic targets. We found that using k-mers as the genetic variant instead of unitigs did not affect the results. The significant k-mers with the largest effect on penicillin susceptibility (p=5·3 × 10^−14^, β −2·5) overlapped the *penA_*01 unitig, and the significant k-mers with the largest effect on tetracycline susceptibility (p=4·4×10^−16^, β −1·6) overlapped the wildtype *rpsJ* 57 unitig.

We used the presence of *penA*_01 combined with the absence of *bla*_TEM_ to predict penicillin susceptibility in our global dataset (figure). We found that this susceptibility-associated genotype predicted penicillin susceptibility and non-resistance with high specificity (99·8%) and modest sensitivity (36·7%) (table). For tetracycline susceptibility prediction, we identified isolates with the wildtype allele at *rpsJ* codon 57 combined with the absence of *tetM* (figure). This combination predicted tetracycline susceptibility and non-resistance with high specificity (97·2%) and sensitivity (88·7%; table). The addition of one chromosomal marker improves performance, as prediction of susceptibility based on plasmid-encoded determinants alone had low sensitivity in our dataset (appendix p 8).

Since penicillin and tetracycline MICs were not reported for all isolates, we identified these mutations in our full genomic dataset: 252 (2·1%) of 12 045 isolates had the penicillin susceptibility-associated genotype, and 1951 (15·9%) of 12 045 isolates had the tetracycline susceptibility-associated genotype. The prevalence of these genotypes varied across genomic epidemiology studies (appendix p 9). Most isolates with non-susceptible genotypes encode only chromosomal resistance deter minants. Among isolates with penicillin non-susceptible genotypes, 1734 (14·7%) of 11 793 encoded *bla*_TEM_. 1636 (19·3%) of 8491 isolates with tetracycline non-susceptible genotypes encoded *tetM*.

To validate our observations in a relatively unbiased dataset from the USA, we assembled a published collection of *N gonorrhoeae* genomes from CDC’s GISP.^18^ In this collection, isolates were not selected for sequencing based on their susceptibility phenotypes. First, we verified that the *penA* sequence identified in the GWAS (*penA_*01) also identified isolates with non-mosaic *penA* alleles without the 345 insertion in the validation dataset. In this dataset, all 57 isolates with *penA_*01 encoded non-mosaic *penA* alleles without the insertion when the full length *penA* allele was examined.

We also calculated sensitivity and specificity for the prediction of penicillin and tetracycline susceptibility and non-resistance in the GISP collection (figure, table). In two isolates, we were unable to genotype *rpsJ* codon 57 because of insufficient coverage of either the reference or alternate allele. Similar to results from the global collection, specificity was high for both antibiotics and CLSI cutoffs. Sensitivity increased for penicillin prediction and decreased for tetracycline prediction, reflecting different proportions of isolates with MICs at the CLSI breakpoints in the global and validation datasets compared with the number of true positives in the dataset. For example, 151 (88·3%) of 171 false negative isolates in the global dataset have MICs at the breakpoint of 0·06 μg/mL, and the global dataset contains a lower proportion of susceptible isolates, with only 99 true positives (appendix p 10).

In addition to antimicrobial resistance phenotypes, GISP reports information on patient characteristics for each isolate collected. To analyse the utility of these genotypic markers in different patient populations, we calculated the prevalence of the susceptibility-associated genotypes across patient groups. Susceptible genotypes were more common among men who have sex with women (MSW) compared to men who have sex with men (MSM) and men who have sex with men and women (MSMW) for penicillin (χ^2^ test, df=3, p=0·0035) and tetracycline (χ^2^ test, df=3, p<0·0001). The prevalence of the penicillin susceptibility-associated genotype was 5·2% (44 of 853 isolates) in MSW, 1·5% (seven of 479) in MSM, and 2·2% (two of 91) in MSMW. For tetracycline, the susceptibility-associated genotype was 20·6% in MSW (175 of 851), 9·6% in MSM (46 of 479), and 9·9% (nine of 91) in MSMW. Additionally, the susceptibility-associated genotypes varied across race and ethnicity groups and were enriched in samples from Black men; however, prevalence of susceptibility-associated genotypes did not differ between race and ethnicity groups when MSM and MSW were considered separately (appendix p 11).

## Discussion

The findings of this genome-wide association study incorporating known, high effect size variants^20^ to identify targets for plasmid and chromosomally mediated penicillin and tetracycline resistance showed that the combination of *penA*_01 (representing non-mosaic *penA*^9^ without an insertion at codon 345^10^) and the absence of *bla*_TEM_ predicts penicillin susceptibility, and that the combination of *rpsJ* codon 57^8^ and the absence of *tetM* predicts tetracycline susceptibility. These loci defined the most susceptible isolates in our dataset and predicted susceptibility (penicillin MIC ≤0·06 μg/mL, tetracycline MIC ≤0·25 μg/mL) with high specificity to both antibiotics in our global dataset and in an unbiased collection from the USA. Sensitivity was high for tetracycline susceptibility prediction and modest for penicillin susceptibility prediction.

Given that many gonorrhoea infections are diagnosed by molecular tests and culture and subsequent MIC testing requires multiple days, gonorrhoea infections are currently treated empirically based on population levels of resistance. Point-of-care diagnostics are a potential approach for targeted therapy of gonorrhoea in the future. Our results suggest that, of the many possible chromosomal loci to predict penicillin and tetracycline susceptibility, *penA*_01 and *rpsJ* are promising targets for diagnostic development. Given that currently available molecular diagnostics (including SpeeDx ResistancePlus GC^5^ and Xpert MTB/RIF^25^) target multiple loci, we expect that a diagnostic tool incorporating the loci identified here, in addition to *gyrA* 91 (comprising five total loci), could be developed using existing technology to provide susceptibility information for three antibiotics. These loci could additionally be used for culture-independent molecular epidemiology and surveillance, as whole-genome sequencing directly from patient samples is not currently routine. Typing schemes, such as NG-STAR,^26^ targeting resistance determinants have been developed; however, these schemes have not focused on loci specific to penicillin and tetracycline resistance.

Utility of a diagnostic or molecular surveillance targeting these loci might vary in different patient populations. For example, the prevalence of susceptibility associated genotypes varied across genomic epidemiology studies included in our global dataset, reflecting both enrichment of antibiotic resistant isolates in some studies and variable selection pressure from antibiotic use in different regions. Whole-genome sequencing data from *N gonorrhoeae* isolated in the USA, Europe, and Australia account for the majority of available genomic data, and the composition of the *N gonorrhoeae* population in other regions is unknown. Similar to other studies of the association between *N gonorrhoeae* antibiotic resistance and patient demographics, prevalence of these susceptibility-associated genotypes vary across patient groups defined by sexual behaviour and race or ethnicity in isolates collected by GISP.^27–29^ In the USA, a diagnostic for penicillin and tetracycline susceptibility might be most useful in populations with increased prevalence of infection with susceptible isolates, such as MSW and women.

In addition to the uneven sampling mentioned above, our study has two key limitations. Although we assigned isolates as susceptible based on MIC, MIC measurements can vary by up to two doubling dilutions, which makes the categorisation of isolates with MICs near the breakpoint potentially more prone to error. However, errors of this magnitude are rare.^30^ We focused on identifying a single chromosomal locus to combine with the absence of plasmid-encoded determinants and predict susceptibility. The addition of other loci (eg, *mtr* and *porB*) could be needed to increase sensitivity for the higher cutoff (MIC <2 μg/mL), but the effect of this on specificity is currently unclear.

In summary, the alleles we have identified from genomic analyses are promising targets for the development of point-of-care molecular diagnostics for *N gonorrhoeae* susceptibility to penicillin and tetracycline. Diagnostics that evaluate as few as two loci per drug could allow for the reintroduction into clinical use of these gonococcal treatment regimens. The effect of test sensitivity on treatment options and prevalence of antibiotic resistance and the effect of querying additional loci are important avenues for future research and further development of sequence-based diagnostics of antimicrobial susceptibility.

## Supplementary Material

1

## Figures and Tables

**Figure:**
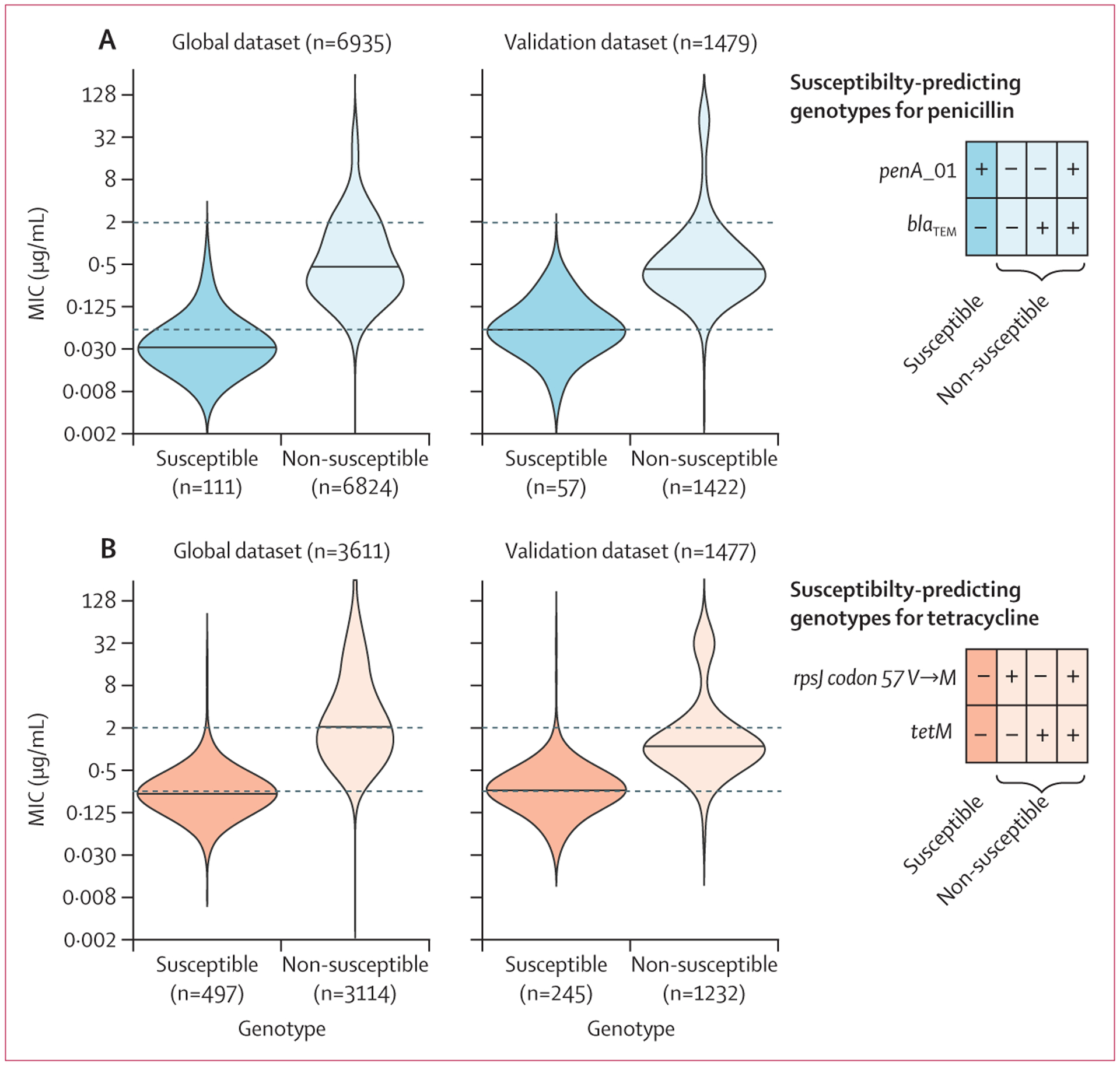
Penicillin (A) and tetracycline (B) MICs in isolates with susceptibility-associated genotypes, global and validation datasets Dashed lines indicate Clinical & Laboratory Standards Institute breakpoints for susceptibility and resistance. (A) Penicillin MICs of isolates with *penA*_101 and without *blaTEM* (susceptible genotype) compared with isolates with one or more of these determinants (non-susceptible genotypes). (B) Tetracycline MICs of isolates with wildtype *rpsJ* (57V) and without *tetM* (susceptible genotype) compared with isolates with one or more of these determinants (non-susceptible genotypes). MIC=minimum inhibitory concentration.

**Table:** **Sensitivity and specificity of genotypes for predicting PCN and TET susceptibility**)

	Global dataset		Validation dataset (GISP 12018^18^)	
	Sensitivity (95% CI)	Specificity (95% CI)	Sensitivity (95% CI)	Specificity (95% CI)
***penA*_01 without *bla*_*TEM*_**				
PCN susceptible (MIC ≤0.06 μg/mL)	36·7% (27·4–46·5)	99·8% (99·0–100·0)	63·6% (49·1–78·2)	98·9% (95·8–100·0)
PCN non-resistant (MIC <2 μg/mL)	2·1% (0·1–4·7)	100·0% (100·0–100·0)	4·4% (0·0–9·8)	100·0% (100·0–100·0)
***rpsJ* WT without *tetM***				
TET susceptible (MIC ≤0.25 μg/mL)	88·7% (86·0–92·1)	97·2% (95·6–98·8)	78·2% (72·2–84·2)	94·9% (91·7–98·1)
TET non-resistant (MIC <2 μg/mL)	28·3% (24·2–32·2)	99·7% (99·2–100·0)	22·1% (16·9–27·3)	99·5% (98·6–100·0)

MIC=minimum inhibitory concentration. PCN=penicillin. TET=tetracycline.

## Data Availability

The analysis pipeline and data are available on GitHub.

## Research in context

### Evidence before this study

We searched PubMed twice for reports published in any language between database inception and July 1, 2021, with the search terms (1) “*Neisseria gonorrhoeae*” and “diagnostic” or “assay” plus “penicillin” or “tetracycline” and (2) “*Neisseria gonorrhoeae*” and “genome wide association study”. We found that previously proposed molecular diagnostics for penicillin and tetracycline susceptibility either exclusively focused on plasmid-mediated resistance (ie, targeting *bla*_TEM_ or *tetM*) or did not include variants in genes encoding antibiotic targets (eg, did not include *penA* or *rpsJ*). Targets for molecular surveillance have focused on resistance-associated alleles rather than susceptibility-associated alleles. We did not find any previous penicillin or tetracycline conditional genome-wide association studies (GWAS) in *N gonorrhoeae*.

### Added value of this study

To identify targets for molecular diagnostics that predict penicillin and tetracycline susceptibility, we conducted GWAS conditioning on the presence of plasmid-mediated resistance determinants to detect chromosomal loci with the highest association with susceptibility. We discovered a sequence (*penA*_01) that differentiates susceptible isolates from those with a resistance-associated insertion at codon 345 and from those with mosaic *penA* alleles, which is associated with penicillin susceptibility. We also found that *rpsJ* codon 57 was the chromosomal locus contributing the most to tetracycline susceptibility. The combination of these chromosomal loci and the absence of plasmid-encoded determinants predicts penicillin and tetracycline susceptibility with high specificity in both a large global collection of *N gonorrhoeae* and a validation dataset consisting of recently published genomes from the US Centers for Disease Control and Prevention’s Gonococcal Isolate Surveillance Program surveillance collected in 2018.

### Implications of all the available evidence

The chromosomal loci *penA_*01 and *rpsJ* codon 57 in combination with plasmid loci *bla*_TEM_ and *tetM* are candidates for the development of point-of-care molecular diagnostics for penicillin and tetracycline susceptibility. The loci could be combined with the currently available ciprofloxacin susceptibility diagnostics to predict susceptibility to multiple antibiotics. Additionally, our study suggests that conditional GWAS focused on variants associated with susceptibility might be a promising approach to identify minimal sets of loci for molecular diagnostics and surveillance.
